# Physical activity and renal cell carcinoma among black and white Americans: a case-control study

**DOI:** 10.1186/1471-2407-14-707

**Published:** 2014-09-24

**Authors:** Qian Xiao, Linda Liao, Charles E Matthews, Wong-Ho Chow, Faith Davis, Kendra Schwartz, Mark Purdue, Jonathan N Hofmann, Joanne Colt

**Affiliations:** Division of Cancer Epidemiology and Genetics, Department of Health and Human Services, National Cancer Institute, National Institutes of Health, Bethesda, MD USA; Department of Epidemiology, The University of Texas MD Anderson Cancer Center, Houston, Texas USA; School of Public Health, University of Alberta, Alberta, Canada; Karmanos Cancer Institute and Department of Family Medicine & Public Health Sciences, Wayne State University, Detroit, MI USA

**Keywords:** Renal cell carcinoma, Physical activities, Racial differences, Case-control study

## Abstract

**Background:**

Renal cell carcinoma (RCC) has a higher incidence in blacks than in whites. Physical activity may influence the risk of renal cell cancer, but the evidence is inconsistent. No previous study has investigated this relationship in the black population.

**Methods:**

We examined the association between self-reported physical activity at different ages and risk of RCC in a population based case-control study of 1217 cases (361 black, 856 white) and 1235 controls (523 black, 712 white) frequency-matched on age, race, and gender. Multivariate-adjusted odds ratios (OR) and 95% confidence intervals (CI) were estimated using unconditional logistic regression.

**Results:**

Among whites, increased risks of RCC were observed among participants reporting low levels of transportation-related activity in their 20’s (OR _<1 hr/wk vs >7 hr/wk_ (95% CI): 1.42 (1.10, 1.83)) and leisure time activity in their 50’s (OR _<1 hr/wk vs >7 hr/wk_ (95% CI): 1.49 (1.00, 2.20)). We found no association between physical activity and RCC risk among blacks.

**Conclusion:**

Our results suggest that physical activity may be inversely associated with RCC risk in whites, but there was no evidence of such an association in blacks. As this is the first study evaluating the effect of physical activity on RCC risk among blacks, further investigations are needed to clarify the relationship in this population.

**Electronic supplementary material:**

The online version of this article (doi:10.1186/1471-2407-14-707) contains supplementary material, which is available to authorized users.

## Background

Kidney Cancer, the deadliest form of urologic cancer, is estimated to have been diagnosed among 40,430 men and 24,720 women in the United States in 2013 [1]. The incidence of renal cell carcinoma (RCC), the major subtype that accounts for ~90% of all kidney cancers, has been increasing rapidly in U.S. over the past three decades [2]. Established modifiable risk factors for RCC include smoking, hypertension, and obesity [3].

It has been postulated that physical activity may protect against RCC by reducing obesity, lowering blood pressure and improving insulin sensitivity [4]. Some studies found an inverse relationship between some aspect of physical activity and RCC risk [5–12], yet others found null associations [13–22]. A recent meta-analysis summarized the findings of previous studies and concluded that a high level of physical activity is associated with a modest reduction in RCC risk (RR (95% CI): 0.88 (0.79-0.97)) [4]. Several studies also examined physical activity of different types or at different ages, but their findings were not consistent [5, 6, 10, 11, 16, 17].

Racial disparities in RCC have been observed. Black Americans have experienced a more rapid increase in incidence in recent decades compared to white Americans, and the incidence is currently 10-15% higher among blacks than among whites [3]. Black RCC patients also have a poorer 5-year survival vs. white patients (73% vs. 68%) [23]. Besides two Asian studies [12, 22], all previous investigations of physical activity and RCC risk were conducted in predominantly Caucasian populations, and we are not aware of any study that reported race-specific results in black populations. The Kidney Cancer Study, one of the largest epidemiologic studies of RCC in the United States, is the first to enroll a sizable number of black Americans, enabling studies of risk factors in this racial group. Here, we investigate race-specific associations of different types of physical activity, at different ages, with RCC risk.

## Methods

### Study population

The Kidney Cancer Study is a population-based case-control study conducted between 2002 and 2007 in Detroit, Michigan (Wayne, Oakland and Macomb Counties) and Chicago, Illinois (Cook County). Study procedures were approved by Institutional Review Boards at National Cancer Institute, University of Illinois at Chicago, Wayne State University, and Westat, Inc. Written informed consent was obtained from all subjects. Details of the study have been described before [24]. Briefly, blacks and whites between 20-79 years of age with an incident, histologically-confirmed diagnosis of RCC (RCC) (ICD-O-3 C64.9) during the enrollment period were eligible to participate. Controls were selected from the general population and frequency matched to cases on sex, age (5-year intervals) and race. In order to recruit a sufficient number of African Americans, we devised a sampling strategy aimed at enrolling all eligible black cases, but only a subsample of white cases. In addition, the control-to-case ratio was targeted at 2:1 for blacks and 1:1 for whites [24]. Histologic subtypes were determined by expert renal pathologist review or based on information from the original diagnostic pathology reports.

Details on recruitment and exclusion have been reported before [24]. Briefly, of 1,918 eligible cases identified, 347 were not contacted due to death, lack of current location or physician refusal to give permission. Among the remaining 1,571 cases, 221 declined participation and 133 were not interviewed due to serious illness, impairment, or nonresponse after multiple contact attempts. Of 2,718 eligible controls, 449 were not contacted due to death or lack of current location. Among the remaining 2,269 controls, 677 declined participation and 357 were not interviewed due to serious illness, impairment, or lack of response to multiple contact attempts. In total, 1,217 cases and 1,235 controls eventually participated.

### Assessment of physical activity

Computer-assisted personal interviews were conducted in the participants’ homes by trained interviewers. Participants were asked to report, for the years they were in their 20’s and 50’s, the amount of time (<1 hr/wk, 1-7 hr/wk, >7 hr/wk, don’t know) spent on physically active transportation (walking or bicycling) or moderate-to-strenuous leisure time activities. Participants who had a full-time or part-time job in their 20’s or 50’s were asked to report the time (<1 hr/wk, 1-10 hr/wk, 11-20 hr/wk, >20 hr/wk and don’t know) spent “doing work that involved moderate to strenuous activity, such as brisk walking, heavy lifting, digging or heavy construction”, at these ages. Participants younger than 23 were skipped from the section on physical activity in their 20’s and participants younger than 53 did not answer questions about activities in their 50’s. Participants who were 23-31 or 53-61 years old at interview were asked to exclude the two years preceding the interview when answering these questions. In total 2,443 and 1,759 participants answered at least one of the physical activity questions for their 20’s and 50’s, respectively.

We created an index of total physical activity at different ages. We assigned a numeric value to each duration category of physical activity. For both transportation and leisure-time activity, we assigned the value of 1, 2, and 3 to the three categories, from the lowest to the highest. For the four categories of work activity, 1, 2, 3, and 4 were assigned. The physical activity scores from all questions at the two ages were calculated by summing up the three different types of activity.

We also collected information on demographic characteristics, BMI at 5 years before recruitment (henceforth referred to as usual BMI) and BMI at age 21, diet, smoking, alcohol drinking, and medical history.

### Statistical analysis

Odds ratios (ORs) and 95% confidence intervals (CIs) were calculated using unconditional logistic regression using STATA 12.0 (StataCorp LP, TX). As described before [24], sample weights were created to reduce the potential for bias caused by differential sampling rates for controls and cases, survey nonresponse, and deficiencies in coverage of the population. In the weighted multivariate regressions, we adjusted for study center (Detroit or Chicago), age (20-44, 45-54, 55-64, 65-74, 75+ years), sex, education (<12 years, high school, some college, 4+ years of college), smoking status (never, occasional, former, current), and history of cancer among first-degree relatives (none, cancer other than kidney cancer, kidney cancer). Further adjustment for alcohol drinking led to minimal changes in the results and therefore we excluded alcohol from the models. BMI and hypertension were considered as potential mediators, and additional adjustment for these factors had minimal impact on the results; therefore they were not included in the models. We performed subgroup analyses by sex, BMI and hypertension. We also conducted sensitivity analysis by excluding cases that were not clear cell subtype. To test for trend, we modeled categorical variables as continuous and evaluated the coefficient using the Wald test. Statistical significance for interactions between two factors was tested using the likelihood ratio test comparing a model with the cross-product term to one without.

## Results

Selected characteristics of participants by race and case-control status are presented in Table 1. In both blacks and whites, when compared to controls, cases were more likely to have <12 years of education, to be obese (BMI ≥30), to have a history of hypertension, and to be current smokers (all p-values for chi-sq test were <0.05 except for black smokers).

**Table 1:** **Selected characteristics of study participants by race and case**-**control status**

	Black		White	
Variable, N (weighted %)	Controls (N = 523)	Cases (N = 361)	Controls (N = 712)	Cases (N = 856)
Study Site				
Chicago	96 (21)	81 (21)	101 (16)	118 (15)
Detroit	427 (79)	280 (79)	611 (84)	738 (85)
Age, years				
20-44	86 (12)	41 (12)	93 (10)	106 (10)
45-54	125 (26)	102 (26)	145 (20)	185 (20)
55-64	145 (30)	117 (30)	205 (29)	255 (26)
65-74	133 (24)	82 (24)	196 (28)	221 (28)
75+	34 (8)	19 (8)	73 (13)	89 (13)
Sex				
Male	250 (61)	225 (61)	439 (62)	495 (62)
Female	273 (39)	136 (39)	273 (38)	361 (38)
Education				
<12 years	100 (19)	97 (28)	65 (9)	103 (13)
High school graduate	176 (34)	104 (29)	214 (31)	315 (37)
Some college	172 (32)	113 (30)	184 (26)	215 (25)
4+ years college	75 (15)	47 (13)	249 (35)	223 (26)
Body mass index, kg/m2				
<25	150 (28)	68 (19)	216 (29)	172 (19)
25- < 30	199 (40)	126 (36)	294 (42)	310 (37)
30- < 35	95 (19)	88 (24)	126 (18)	210 (25)
35+	73 (13)	74 (20)	74 (10)	156 (17)
Missing	6 (1)	5 (1)	2 (<1)	8 (1)
Smoking satus				
Never	184 (34)	123 (34)	287 (40)	309 (36)
Occasional	30 (5)	21 (6)	25 (3)	34 (4)
Former	169 (34)	106 (29)	276 (39)	304 (37)
Current	140 (26)	111 (31)	124 (17)	209 (23)
Family history of cancer				
None	288 (54)	183 (51)	278 (38)	334 (38)
Cancer other than kidney	220 (44)	152 (42)	413 (59)	484 (57)
Kidney cancer	9 (1)	19 (5)	15 (2)	33 (4)
Missing	7 (2)	6 (1)	6 (1)	5 (1)
Hypertension				
Yes	246 (48)	256 (71)	262 (38)	445 (54)
No	273 (51)	102 (29)	445 (61)	398 (44)
Missing	4 (1)	3 (<1)	5 (1)	13 (2)

In Table 2 we summarize the association of RCC with transportation, leisure time, work and total physical activities in blacks and whites. In whites, low levels of transportation-related activity in their 20’s and leisure time activity in their 50’s were associated with increased odds of RCC. Compared to the reference group (>7 hr/week of activities), whites who reported engaging in these activities for <1 hr/week were >40% more likely to be diagnosed with RCC [OR (95%): 1.42 (1.10, 1.83) for transportation activity in their 20’s and 1.49 (1.00, 2.20) for leisure time activity in their 50’s]. No association with renal cancer was found for work activity at either age. Additionally, there was a suggestive inverse, albeit statistically nonsignificant, association between total activity score both in their 20’s and 50’s and renal cancer in whites. In blacks, neither the individual types of activity nor the total activity score at any age was associated with renal cancer. There were no statistically significant interactions between race and any of the physical activity measures. After restricting our analysis to clear-cell subtype, the results remained largely similar (Additional file 1: Table S1).

**Table 2:** **Renal cell carcinoma in relation to physical activity at age 20**’**s and 50**’**s**, **by race**

	Black			White			***p for interaction***
	Controls, no. (%) ^a^	Cases, no. (%) ^a^	Odds ratio (95% CI) ^b^	Controls, no. (%) ^a^	Cases, no. (%) ^a^	Odds ratio (95% CI) ^b^	
Physical activity during age 20’s							
Walking or biking for transportation, hr/wk							*0.17*
<1	122 (24)	89 (25)	1.06 (0.73, 1.53)	232 (32)	335 (39)	1.42 (1.10, 1.83)	
1-7	150 (29)	102 (28)	1.00 (0.72, 1.38)	273 (40)	307 (36)	1.06 (0.80, 1.39)	
>7	243 (47)	165 (46)	ref	198 (28)	212 (25)	ref	
*p trend*			*0.77*			*0.01*	
Moderate to strenuous leisure time activity, hr/wk							*0.57*
<1	94 (16)	65 (18)	1.16 (0.79, 1.71)	100 (14)	134 (16)	1.10 (0.81, 1.48)	
1-7	193 (35)	131 (37)	1.10 (0.79, 1.53)	352 (50)	412 (48)	0.98 (0.78, 1.25)	
>7	230 (48)	161 (44)	ref	155 (36)	307 (36)	ref	
*p trend*			*0.41*			*0.65*	
Moderate to strenuous work activity, hr/wk							*0.68*
<1	119 (19)	65 (19)	1.07 (0.68, 1.67)	232 (34)	283 (32)	1.05 (0.81, 1.36)	
1-10	95 (18)	79 (21)	1.18 (0.82, 1.70)	134 (19)	159 (19)	1.05 (0.74, 1.48)	
11-20	53 (11)	29 (9)	0.75 (0.44, 1.30)	88 (12)	73 (9)	0.71 (0.50, 1.02)	
>20	202 (43)	155 (43)	ref	208 (29)	271 (32)	ref	
*p trend*			*0.96*			*0.45*	
Total activity score^c^							*0.07*
2-4	80 (13)	42 (12)	1.14 (0.68, 1.92)	118 (17)	168 (19)	1.35 (0.91, 2.01)	
5-6	135 (24)	99 (28)	1.37 (0.94, 2.00)	243 (35)	297 (35)	1.16 (0.84, 1.63)	
7-8	141 (31)	113 (32)	1.24 (0.84, 1.82)	236 (34)	266 (32)	0.97 (0.71, 1.33)	
9-10	159 (32)	99 (28)	ref	103 (14)	119 (15)	ref	
*p trend*			*0.30*			*0.06*	
Physical activity during age 50’s							
Walking or biking for transportation, hr/wk							*0.17*
<1	134 (38)	95 (36)	0.78 (0.50, 1.22)	249 (47)	305 (48)	0.98 (0.67, 1.45)	
1-7	133 (39)	96 (37)	0.79 (0.51, 1.24)	212 (41)	235 (38)	0.90 (0.60, 1.34)	
>7	78 (22)	68 (27)	ref	66 (13)	80 (13)	ref	
*p trend*			*0.31*			*0.83*	
Moderate to strenuous leisure time activity, hr/wk							*0.21*
<1	80 (24)	61 (23)	0.84 (0.50, 1.43)	72 (13)	120 (19)	1.49 (1.00, 2.20)	
1-7	159 (43)	113 (44)	1.06 (0.68, 1.65)	295 (55)	316 (50)	0.95 (0.72, 1.25)	
>7	107 (32)	85 (32)	ref	158 (32)	185 (30)	Ref	
*p trend*			*0.58*			*0.09*	
Moderate to strenuous work activity, hr/wk							*0.71*
<1	98 (27)	60 (23)	0.86 (0.56, 1.32)	197 (37)	226 (36)	0.98 (0.73, 1.31)	
1-10	67 (19)	55 (21)	1.19 (0.70, 2.01)	122 (23)	111 (18)	0.72 (0.50, 1.04)	
11-20	36 (12)	17 (6)	0.53 (0.28, 1.01)	51 (10)	53 (8)	0.79 (0.50, 1.23)	
>20	115 (34)	86 (34)	ref	121 (22)	159 (26)	ref	
*p trend*			*0.89*			*0.88*	
Total activity score^c^							*0.14*
2-4	82 (22)	63 (24)	1.03 (0.58, 1.84)	133 (24)	180 (28)	1.50 (0.95, 2.41)	
5-6	104 (31)	53 (33)	1.01 (0.57, 1.77)	220 (40)	222 (36)	1.21 (0.76, 1.92)	
7-8	102 (30)	66 (26)	0.93 (0.55, 1.57)	142 (27)	171 (29)	1.29 (0.80, 2.10)	
9-10	55 (17)	44 (17)	ref	48 (9)	45 (8)	ref	
*p trend*			*0.79*			*0.12*	

^a^Percentages are weighted.

^b^Adjusted for study center (Detroit or Chicago), age (20-44, 45-54, 55-64, 65-74, 75+ years), sex (male, female), education (<12 years, high school, some college, 4+ years of college), smoking status (never, occasional, former, current), and history of cancer among first-degree relatives (none, cancer other than kidney cancer, kidney cancer, unknown).

^c^Numeric scores were assigned to each category of physical activities (Transportation and leisure activities: 1, 2, and 3 for <1, 1-7 and >7 hr/wk, respectively; work activity: 0, 1, 2, 3, and 4 for none, <1, 1-10, 11-20 and >20 hr/wk, respectively). Total scores were calculated by combining all the activity scores of each person at different age periods.

In these analysis, we excluded people with missing information on specific types of physical activity (transportation: N = 15 for age 20’s and N = 8 for age 50’s; leisure-time: N = 9 for age 20’s and N = 8 for age 50’s; work: N = 7 for age 20’s and N = 4 for age 50’s). Additionally, participants who did not work either full-time or part time were also excluded from the analysis of work activity (N = 191 for age 20’s and 181 for age 50’s).

In subgroup analysis, we did not detect any significant interaction between activity and sex, usual BMI or hypertension status. Among blacks, the association between physical activity and RCC was largely null across subgroups. For physical activity occurring in the 20’s, we also performed subgroup analyses by BMI at age 21, with similar findings (data not shown).

## Discussion

In this large population-based case-control study, we found a suggestive inverse association between physical activity and RCC among whites, but no evidence of an association among blacks. The observed effects were driven mainly by physical activity done outside of the work place, such as walking or biking for transportation and leisure time activities.

The inverse association between physical activity and RCC among whites was consistent with previous studies. A recent meta-analysis [4] summarized 19 studies of predominantly white populations and found a 12% reduction in relative risk of RCC for high total physical activity compared to low physical activity. When they performed stratified analysis by physical activity domains, they found that the RR comparing high vs low recreational activity was 0.88 (95% CI, 0.77-1.00). However, the summary RR for occupational activity from 14 effect estimates was nonsignificant (0.91 (0.79, 1.04)). We also did not find an association between work activity and RCC. The null findings for work-related physical activity may be due to residual confounding, as people who held labor-intensive jobs are more likely to be of low SES, and may have other health risk factors that influence RCC risk.

To our knowledge, this is the first study of physical activity and RCC among blacks. We did not find a statistically significant association between physical activity and RCC among blacks, overall or in subgroup analyses by potential effect modifiers with different prevalence across the two racial groups, such as BMI and hypertension. Few epidemiologic studies have examined risk factors for RCC in the black population [25]. Some risk factors, such as hypertension [24], chronic renal failure [26] and family history of cancer [27], have been found to be positively associated with RCC in both blacks and whites, while others, such as BMI at early age [28], smoking [29] and reproductive factors in women [30], appear to have weaker effects in blacks than whites. Our finding of a lack of association between physical activity and RCC in blacks deserves further exploration.

This study has several limitations. First, our self-reported estimates of past physical activity will have been subject to measurement error, the effects of which may have affected our results. Moreover, the middle categories of transportation and leisure time activity were quite broad and included people ranging from fairly inactive (1 hr/wk) to active (7 hr/wk), making it hard to interpret the effect estimates. Also we lacked of information on physical activities between age 20’s to 50’s, and were not able to examine its relationship with renal cancer. Although we adjusted for potential confounders, we could not rule out the possibility of residual confounding. If the level of residual confounding differs by race, it would make direct comparison between blacks and whites problematic. We have performed multiple comparisons and the findings of inverse association among whites can be due to chance alone. Lastly, we had a fairly low response rate among controls compared to cases. The sample weights used in our analysis are designed to account for differential nonresponse across subgroups defined by factors such as age, sex, and county of residence, and can partially reduce bias. However, it is still possible that the nonresponse rate may differ by physical activity level, which can lead to biased estimates.

A notable strength of our study is that by oversampling African Americans, we were able to assess the relationship between physical activity and RCC in blacks and make comparisons between the two races. We also had a sufficient sample size to conduct subgroup analysis by several potential effect modifiers such as sex, BMI and hypertension.

## Conclusions

In summary, our findings suggest that low levels of physical activity may increase the risk of RCC in whites. In contrast, higher levels of physical activity did not appear to offer similar protective effect in blacks.

## Electronic supplementary material

Additional file 1: Table S1: Clear Cell Renal Cell Carcinoma in Relation to Physical Activity at Age 20’s and 50’s, by Race. (DOC 74 KB)

## Abbreviations

BMI: Body mass index
CI: Confidence interval
OR: Odds ratio
RCC: Renal cell carcinoma.
