# Supplementary material for: Physical activity and renal cell carcinoma among black and white Americans: a case-control study
Source: BMC Cancer. 2014 Sep 24;14:707. doi: 10.1186/1471-2407-14-707 (PMC4181698; doi:10.1186/1471-2407-14-707)
Supplement: Supplementary file 1 — Additional file 1: Table S1: Clear Cell Renal Cell Carcinoma in Relation to Physical Activity at Age 20’s and 50’s, by Race. (DOC 74 KB) [file 12885_2014_4879_MOESM1_ESM.doc]

**Additional file Table 1** Clear Cell Renal Cell Carcinoma in Relation to Physical Activity at Age 20’s and 50’s, by Race

|  | **Black** | | | **White** | | | *p for interaction* |
| --- | --- | --- | --- | --- | --- | --- | --- |
|  | **Controls, no. (%) ^a^** | **Cases, no. (%) ^a^** | **Odds Ratio (95% CI) ^b^** | **Controls, no. (%) ^a^** | **Cases, no. (%) ^a^** | **Odds Ratio**  **(95% CI) ^b^** |
| Physical activity during age 20's | | | | | | | |
| Walking or biking for transportation, hr/wk | | | | | | | *0.20* |
| <1 | 122 (24) | 39 (24) | 1.05 (0.65, 1.72) | 232 (32) | 220 (40) | 1.46 (1.11, 1.93) |  |
| 1-7 | 150 (29) | 49 (30) | 1.05 (0.69, 1.60) | 273 (40) | 186 (34) | 1.00 (0.72, 1.38) |  |
| >7 | 243 (47) | 77 (46) | ref | 198 (28) | 136 (25) | ref |  |
| *p trend* |  |  | *0.81* |  |  | *0.005* |  |
| Moderate to strenuous leisure time activity, hr/wk | | | | |  |  | *0.83* |
| <1 | 94 (16) | 27 (17) | 1.02 (0.59, 1.75) | 100 (14) | 88 (16) | 1.09 (0.78, 1.51) |  |
| 1-7 | 193 (35) | 59 (36) | 1.02 (0.68, 1.55) | 352 (50) | 255 (46) | 0.92 (0.72, 1.19) |  |
| >7 | 230 (48) | 78 (47) | ref | 155 (36) | 200 (38) | ref |  |
| *p trend* |  |  | *0.93* |  |  | *0.86* |  |
| Moderate to strenuous work activity, hr/wk | | | |  |  |  | *0.14* |
| <1 | 119 (19) | 35 (25) | 1.42 (0.81, 2.47) | 232 (34) | 184 (36) | 1.14 (0.84, 1.55) |  |
| 1-10 | 95 (18) | 37 (23) | 1.32 (0.78, 2.23) | 134 (19) | 91 (19) | 1.01 (0.67, 1.52) |  |
| 11-20 | 53 (11) | 11 (8) | 0.68 (0.29, 1.63) | 88 (12) | 47 (10) | 0.79 (0.51, 1.22) |  |
| >20 | 202 (43) | 65 (43) | ref | 208 (29) | 167 (35) | ref |  |
| *p trend* |  |  | *0.14* |  |  | *0.32* |  |
| Total activity score ^c^ | | | | | | | *0.63* |
| 2-4 | 80 (13) | 20 (13) | 1.35 (0.64, 2.81) | 118 (17) | 113 (20) | 1.46 (0.96, 2.23) |  |
| 5-6 | 135 (24) | 48 (29) | 1.70 (1.02, 2.84) | 243 (35) | 191 (35) | 1.29 (0.82, 1.73) |  |
| 7-8 | 141 (31) | 55 (33) | 1.56 (0.94, 2.60) | 236 (34) | 161 (31) | 0.96 (0.67, 1.37) |  |
| 9-10 | 159 (32) | 40 (25) | ref | 103 (14) | 76 (15) | ref |  |
| *p trend* |  |  | *0.19* |  |  | *0.03* |  |
| Physical activity during age 50's | | | | | | | |
| Walking or biking for transportation, hr/wk | | | | | | | *0.98* |
| <1 | 134 (38) | 45 (38) | 0.71 (0.43, 1.18) | 249 (47) | 196 (48) | 0.92 (0.60, 1.40) |  |
| 1-7 | 133 (39) | 39 (35) | 0.67 (0.38, 1.19) | 212 (41) | 143 (37) | 0.81 (0.52, 1.25) |  |
| >7 | 78 (22) | 30 (27) | ref | 66 (13) | 55 (14) | ref |  |
| *p trend* |  |  | *0.24* |  |  | *1.00* |  |
| Moderate to strenuous leisure time activity, hr/wk | | | | |  |  | *0.43* |
| <1 | 80 (24) | 28 (24) | 0.89 (0.49, 1.64) | 72 (13) | 75 (19) | 1.45 (0.94, 2.23) |  |
| 1-7 | 159 (43) | 49 (44) | 0.99 (0.57, 1.72) | 295 (55) | 199 (50) | 0.94 (0.68, 1.29) |  |
| >7 | 107 (32) | 37 (32) | ref | 158 (32) | 121 (31) | Ref |  |
| *p trend* |  |  | *0.73* |  |  | *0.18* |  |
| Moderate to strenuous work activity, hr/wk | | | |  |  |  | *0.38* |
| <1 | 98 (27) | 27 (26) | 0.96 (0.52, 1.75) | 197 (37) | 150 (42) | 1.03 (0.73, 1.45) |  |
| 1-10 | 67 (19) | 33 (34) | 1.76 (0.95, 3.23) | 122 (23) | 67 (20) | 0.75 (0.49, 1.15) |  |
| 11-20 | 36 (12) | 7 (6) | 0.56 (0.21, 1.51) | 51 (10) | 33 (9) | 0.83 (0.50, 1.38) |  |
| >20 | 115 (34) | 34 (34) | ref | 121 (22) | 94 (28) | ref |  |
| *p trend* |  |  | *0.57* |  |  | *0.89* |  |
| Total activity score  ^c^ | | | | | | | *0.71* |
| 2-4 | 82 (22) | 24 (21) | 0.88 (0.43, 1.80) | 133 (24) | 119 (29) | 1.44 (0.84, 2.48) |  |
| 5-6 | 104 (31) | 44 (38) | 1.08 (0.53, 2.18) | 220 (40) | 145 (37) | 1.19 (0.69, 2.02) |  |
| 7-8 | 102 (30) | 28 (26) | 0.87 (0.47, 1.61) | 142 (27) | 99 (26) | 1.13 (0.65, 1.96) |  |
| 9-10 | 55 (17) | 18 (16) | ref | 48 (9) | 29 (9) | ref |  |
| *p trend* |  |  | *0.95* |  |  | *0.11* |  |

^a^ Percentages are weighted

^b^ Adjusted for study center (Detroit or Chicago), age (20-44, 45-54, 55-64, 65-74, 75+ years), sex (male, female), education (<12 years, high school, some college, 4+ years of college), smoking status (never, occasional, former, current), and history of cancer among first-degree relatives (none, cancer other than kidney cancer, kidney cancer, unknown).

^c^ Numeric scores were assigned to each category of physical activities (Transportation and leisure activities: 1, 2, and 3 for <1, 1-7 and >7 hr/wk, respectively; work activity: 0, 1, 2, 3, and 4 for none, <1, 1-10, 11-20 and >20 hr/wk, respectively). Total scores were calculated by combining all the activity scores of each person at different age periods.

In these analysis, we excluded people with missing information on specific types of physical activity (transportation: N=15 for age 20’s and N=8 for age 50’s; leisure-time: N=9 for age 20’s and N=8 for age 50’s; work: N=7 for age 20’s and N=4 for age 50’s). Additionally, participants who did not work either full-time or part time were also excluded from the analysis of work activity (N=191 for age 20’s and 181 for age 50’s).
